# Diversity change during the rise of tetrapods and the impact of the ‘Carboniferous rainforest collapse’

**DOI:** 10.1098/rspb.2017.2730

**Published:** 2018-02-07

**Authors:** Emma M. Dunne, Roger A. Close, David J. Button, Neil Brocklehurst, Daniel D. Cashmore, Graeme T. Lloyd, Richard J. Butler

**Affiliations:** 1 School of Geography, Earth and Environmental Sciences, University of Birmingham, Birmingham B15 2TT, UK; 2 Paleontology Research Lab, North Carolina Museum of Natural Sciences, 11 W Jones St, Raleigh, NC 27607, USA; 3 Department of Biological Sciences, North Carolina State University, 3510 Thomas Hall, Campus Box 7614, Raleigh, NC 27695, USA; 4 Museum für Naturkunde, Leibniz-Institut für Evolutions- und Biodiversitätsforschung, Invalidenstraße 43, 10115 Berlin, Germany; 5 School of Earth and Environment, University of Leeds, Leeds LS2 9JT, UK

**Keywords:** Tetrapoda, sampling, diversity, biogeography, Carboniferous rainforest collapse, Palaeozoic

## Abstract

The Carboniferous and early Permian were critical intervals in the diversification of early four-limbed vertebrates (tetrapods), yet the major patterns of diversity and biogeography during this time remain unresolved. Previous estimates suggest that global tetrapod diversity rose continuously across this interval and that habitat fragmentation following the ‘Carboniferous rainforest collapse’ (CRC) drove increased endemism among communities. However, previous work failed to adequately account for spatial and temporal biases in sampling. Here, we reassess early tetrapod diversity and biogeography with a new global species-level dataset using sampling standardization and network biogeography methods. Our results support a tight relationship between observed richness and sampling, particularly during the Carboniferous. We found that subsampled species richness initially increased into the late Carboniferous, then decreased substantially across the Carboniferous/Permian boundary before slowly recovering in the early Permian. Our analysis of biogeography does not support the hypothesis that the CRC drove endemism; instead, we found evidence for increased cosmopolitanism in the early Permian. While a changing environment may have played a role in reducing diversity in the earliest Permian, our results suggest that the CRC was followed by increased global connectivity between communities, possibly reflecting both reduced barriers to dispersal and the diversification of amniotes.

## 1. Introduction

Tetrapods (four-limbed vertebrates) first appeared on land in the late Devonian [1,2], and during the Carboniferous and early Permian established the first terrestrial vertebrate communities. In the early Carboniferous, these amphibian-like early tetrapods radiated rapidly and diversified into a wide variety of morphologies and sizes [3]. Later in the Carboniferous, crown amniotes appeared [4], and by the early Permian, the terrestrial vertebrate fauna was dominated by synapsids (the mammalian stem-group), such as edaphosaurids and sphenacodontids, alongside a diverse array of basal reptiles (e.g. captorhinids) and amphibians [5,6].

This diversification occurred as the surrounding environment was transitioning from wetlands in the Carboniferous to more arid conditions in the Permian. During the late Carboniferous, Euramerica (Europe and North America) lay at the equator and was predominantly covered by tropical rainforests, commonly referred to as the ‘Coal Forests’ [7]. During the Kasimovian (approx. 303–307 Ma), these rainforests began to disappear from large parts of the globe, and by the early Permian had been replaced in many regions by dryland vegetation as a more arid climate developed [8,9]. This ‘rainforest collapse’ culminated in what is considered one of two mass extinction events evident in the plant fossil record [10].

Despite this interval being a crucial time for tetrapod evolution and the establishment of terrestrial ecosystems, few studies have focused on Carboniferous–early Permian tetrapod diversity patterns or have attempted to quantify the impact of the ‘Carboniferous rainforest collapse’ (CRC) on the terrestrial vertebrate fauna. Instead, most work has been focused on the later end-Permian mass extinction [11,12] and more recently on the early and mid-Permian extinction events (e.g. [13,14]). A previous study that attempted to assess the impact of the CRC suggested that the newly fragmented habitats following the collapse drove the development of endemism among tetrapod communities [15]. This is proposed to have led to reduced local richness (alpha diversity) but higher global diversity (gamma diversity) following the CRC. However, this study failed to adequately account for how sampling of the fossil record varies in both time and space, largely accepting raw diversity patterns at face value. Moreover, the analysis was conducted using a family-level dataset, rather than one at species level, and some of the data used in this study are no longer accessible.

The impact of uneven sampling on estimates of diversity has been appreciated for almost half a century [16–18], and in recent years there have been an increasing number of studies investigating the influences of sampling biases on palaeodiversity [19–21]. The correlation between palaeodiversity and sampling has been repeatedly demonstrated in many fossil groups, including terrestrial vertebrates [22–25], marine vertebrates [26], insects [27], marine invertebrates [28] and plants [29,30]. Sampling intensity is influenced by several factors including geographical location, volume and variety of preserved sedimentary environments, collection methods and academic interest. Substantial efforts have been made recently to develop statistical methods which can mitigate these biases allowing diversity to be estimated from an incomplete fossil record.

Here, using a newly compiled global species-level dataset alongside sampling standardization and network biogeography methods, we investigate patterns of early tetrapod diversity and biogeography from the Carboniferous to early Permian to answer the following questions. (i) What are the major patterns of tetrapod diversity during this interval? (ii) How do sampling biases impact estimates of diversity, and how can we best account for them? (iii) Did the ‘CRC’ drive the development of endemism among tetrapod communities?

## 2. Material and Methods

Newly compiled data detailing the global occurrences of early tetrapod species from the beginning of the Carboniferous (Tournaisian) to the end of the Cisuralian epoch (Kungurian), informally referred to as the ‘early Permian’, were downloaded from the Paleobiology Database (paleobiodb.org, accessed 19 September 2017). These data result from a concerted effort to document the Palaeozoic terrestrial tetrapod fossil record, led by the lead author of this study. The data represent the current published knowledge on the global occurrences and taxonomic opinions of early tetrapods. Data preparation and analyses were conducted within R v. 3.4.1 [31]. All marine taxa and ichnotaxa were discarded from the dataset, and the final cleaned dataset comprises 476 tetrapod species from 385 collections (= fossil localities), totalling 1047 unique global occurrences.

### (a) Diversity and sampling

To enable direct comparison with earlier studies, we present raw (= uncorrected or observed) diversity patterns at global and local spatial scales. However, we do so with the proviso that raw diversity counts may be highly misleading, and focus on our interpretation of the diversity patterns produced using coverage-based sampling standardization. Global (= gamma scale) raw diversity curves were computed using sampled-in-bin counts of specifically determinate occurrences. Separate curves were computed for (i) all tetrapod species, (ii) non-amniotes (early tetrapodomorphs and amphibians) and (iii) amniotes (including Reptiliomorpha). We also plotted raw family diversity to allow direct comparison with the dataset of previous analyses [15]. Family-level assignments were based upon those recorded in the dynamic taxonomy of the Paleobiology Database.

We estimated local richness (= alpha diversity) by counting species per collection (= fossil locality). These counts included not only occurrences determinate at species level but also those indeterminate at species level that must logically represent distinct species according to the taxonomic hierarchy of the Paleobiology Database.

We focus our interpretation of gamma-scale diversity patterns on coverage-standardized estimates. Coverage-based sampling standardization uses the concept of frequency-distribution coverage (a measure of sample completeness that can be accurately and precisely estimated using Good's *u* [32] to make fair comparisons of diversity between assemblages that may be sampled to very different levels of intensity). Sample coverage is simply the fraction of individuals in the original population that belong to the sampled species (i.e. the degree to which the sampled species ‘cover’ the entire frequency distribution). Alroy [33–36] introduced this method under the name Shareholder Quorum Subsampling (SQS), using an algorithmic approach. However, we chose to implement SQS (also known as ‘coverage-based rarefaction’) using the analytical equations described by Chao & Jost [37] via the R package ‘iNEXT’ (iNterpolation/EXTrapolation) [38]. The analytical implementation of SQS in iNEXT yields confidence intervals and allows coverage-based extrapolation (using the Chao1 estimator), in addition to interpolation (= subsampling). The data were rarefied by collection, by analysing incidence-frequency matrices of the occurrence data. Extrapolated estimates were limited to no more than twice the observed sample size (as recommended by Hsieh *et al*. [38]). We elected not to use the optional three-collections-per-reference protocol advocated by Alroy [36], because (i) unlike marine invertebrate datasets, Carboniferous–early Permian tetrapods do not suffer from over-reporting of common taxa, and (ii) sample coverage in some intervals is so low that limiting the amount of data drawn (to no more than three-collection-per-reference per trial) prohibited us from obtaining diversity estimates at meaningful quorum levels (i.e. target levels of standardized coverage). We computed coverage-standardized diversity estimates at both species and genus level. Both ranked and relative richness among assemblages may change depending on quorum level if there are differences in evenness or the shape of the abundance distribution [37]; therefore, in addition to presenting diversity-through-time curves, we also present coverage-based rarefaction curves to show how coverage-standardized diversity estimates for different time intervals vary with coverage. Additionally, we quantify patterns of sampling using counts of total collections, fossiliferous formations, and occupied equal-area grid cells (50 km spacing) [39] in each interval, and also show how sample-based coverage varies through time using Good's *u* [32,37].

### (b) Phylogenetic biogeographic connectedness

Sidor *et al.* [40] developed a network model of biogeography to assess regional biogeographic changes by quantifying biogeographic connectedness (BC) between regions containing tetrapod fauna. This general approach can be used to test the biogeographic hypothesis proposed by Sahney *et al.* [15] that global tetrapod faunas became increasingly endemic after the CRC (i.e. less well connected). The Sidor *et al.* approach may be of limited utility when analysing a fossil record dominated by ‘singletons’ (taxa occurring at a single locality or within a single geographical area), as is the case for the Carboniferous–early Permian. Instead, we used a modification of the Sidor *et al.* network model presented by Button *et al.* [41] where phylogenetic information is incorporated into the calculation of BC, thus addressing issues arising from using only binary presence–absence data. This method inversely weights links between taxa in different geographical regions in proportion to the phylogenetic distance between them, and these links are used to calculate phylogenetic biogeographic connectedness (pBC). Values of pBC range between 0 and 1, with higher values equating to more cosmopolitan faunas, whereas lower values indicate greater endemism and phylogenetic distinction between geographical regions.

To analyse pBC, we first defined geographical input areas for the analysis through a *k*-means clustering of palaeocoordinate data for all 1047 tetrapod occurrences in the occurrence dataset described above. This approach to defining geographical areas uses only the palaeocoordinate data to identify geographically discrete clusters of fossil localities and does not take into account species relationships or taxonomy. *k*-means clustering was performed within R for each interval separately, varying the value of *k* from 3 to 10. The performance of each iteration (3–10) was compared based upon the percentage of variance explained, and the clusters in each iteration were compared with palaeogeographic reconstructions. This resulted in the designation of seven discrete geographical regions each for the Carboniferous and early Permian. Species were assigned to one or more of the regions as appropriate, creating a taxon-region matrix for each time interval. We assembled an informal species-level supertree of early tetrapods, consisting of 325 species based upon the most recent phylogenetic analyses and formal supertrees available for the major clades of Carboniferous–early Permian tetrapods [42–45]. As in the diversity analyses, marine taxa and taxa indeterminate at the generic and specific levels were excluded. pBC was then calculated for each time interval using the appropriate taxon-region matrix. The constant *μ* was set at 15 million years following Button *et al.* [41]. Jackknifing, with 10 000 replicates, was used to calculate 95% confidence intervals. We performed this analysis first for all tetrapod species in the Carboniferous (Tournaisian–Gzhelian) and early Permian (Asselian–Kungurian), then separately for amphibians and amniotes in the same two intervals, and finally for all tetrapod species in three shorter intervals (pre-CRC, Bashkirian–Kasimovian; immediately post-CRC, Gzhelian–Sakmarian; post-CRC, Artinskian–Kungurian).

## 3. Results and discussion

### (a) Patterns of diversity and sampling

**Figure 1.**
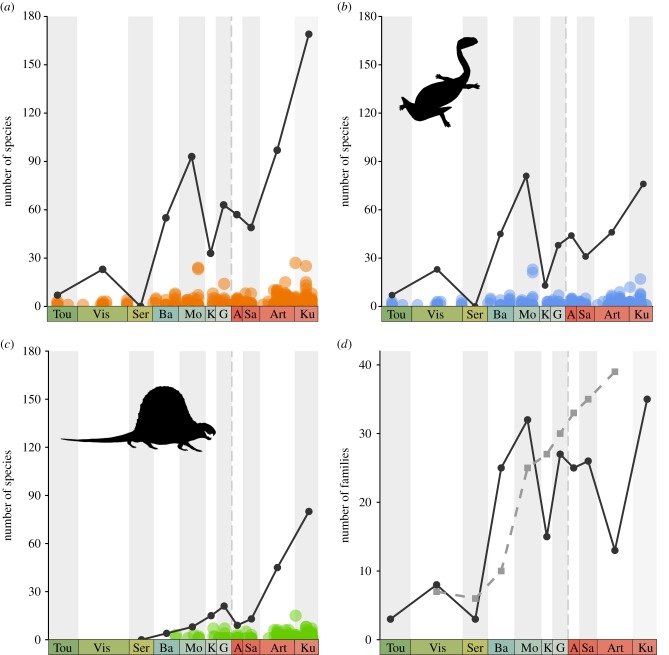
Raw (= uncorrected) richness and local richness (alpha diversity) from the Carboniferous to early Permian. Local richness here is the number of species per collection (= fossil locality). (*a*–*c*) Species richness (solid line) and gamma diversity (circles) for all tetrapod species, non-amniote species and amniote species, respectively. (*d*) Comparison between family diversity estimated by Sahney *et al.* [15] (dashed line) and this study (solid line). Abbreviations of interval names: Tou, Tournaisian; Vis, Visean; Ser, Serpukhovian; Ba, Bashkirian; Mo, Moscovian; K, Kasimovian; G, Gzhelian; A, Asselian; Sa, Sakmarian; Art, Artinskian; Ku, Kungurian. Silhouettes from phylopic.org. (Online version in colour.)

Raw global tetrapod species richness (= uncorrected or ‘observed’ species counts) generally rose from the Carboniferous to early Permian, but this rise was not steady (figure 1*a*). The greatest increases in raw species richness occur during the late Carboniferous (Serpukhovian–Moscovian) and in the final stages of the early Permian (Sakmarian–Kungurian). Carboniferous diversity is dominated by non-amniote taxa (tetrapodomorphs and amphibians), with a marked rise in richness from the Serpukhovian to Moscovian (figure 1*b*). This increase is followed by a substantial decrease in the Kasimovian before richness begins to generally increase again during the early Permian. Amniotes first appeared in the late Carboniferous and from then richness rose into the early Permian, disrupted only by a decrease across the Carboniferous/Permian boundary (Gzhelian–Asselian) (figure 1*c*). By the end of the early Permian, both non-amniotes and amniotes had reached similar levels of species richness. Raw family richness also increased across the interval, as reported by Sahney *et al.* [15]. Directly comparing our estimates of family richness with those of Sahney *et al.* [15] reveal the differences between both datasets (figure 1*d*), which may result in part from the different approach to taxon counting: range-through in Sahney *et al.* [15] (which has the effect of smoothing the diversity curve) and sampled-in-bin counting here.

**Figure 2.**
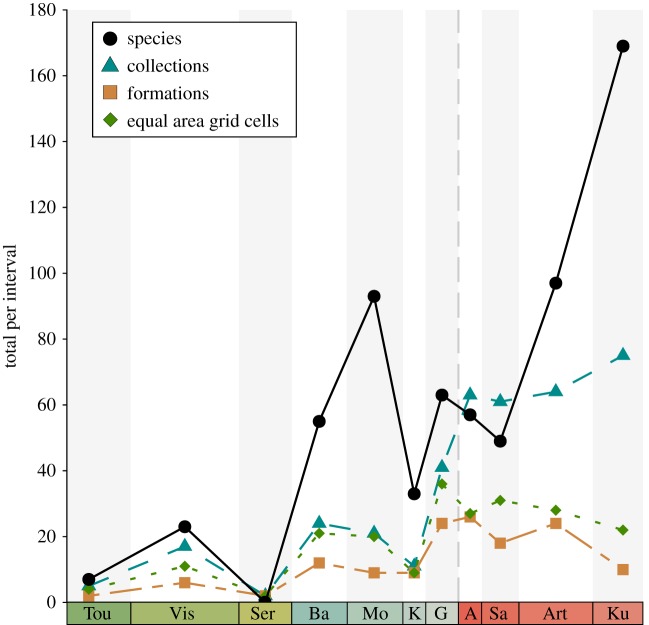
Sampling in the Carboniferous and early Permian. Tetrapod species richness (solid line) closely tracks total number of formations, collections and equal area grid cells in the Carboniferous, but then begins to deviate from this trend in the early Permian. Abbreviations of interval names are as given in figure 1. (Online version in colour.)

Raw species richness estimates are heavily influenced by temporal and spatial sampling biases. From the Carboniferous to early Permian, the numbers of fossiliferous formations, collections (= fossil localities) and occupied equal-area grid cells fluctuate, indicating a high degree of variation in temporal sampling (figure 2 and table 1). Visual inspection shows that raw species richness in the Carboniferous closely tracks patterns of sampling, with intervals where richness is high also having high counts of sampled formations, collections and grid cells (figure 2). In the early Permian, this pattern is less evident, and higher values for Good's *u* in the Asselian, Artinskian and Kungurian indicate that the early Permian is comparatively better sampled than all stages of the Carboniferous (table 1).

**Table 1.** Counts of species, collections (= fossil localities), formations and equal area grid cells in each interval as proxies for sampling. Abbreviations of interval names are as given in figure 1.

	Tou	Vis	Ser	Ba	Mo	K	G	A	Sa	Art	Ku
species	7	23	0	55	93	33	63	57	49	97	169
collections	5	17	2	24	21	11	41	63	61	64	75
formations	2	6	2	12	9	9	24	26	18	24	10
grid cells	4	11	2	21	20	9	36	27	31	28	22
Good's *u*	0.28	0.17	—	0.24	0.33	0.19	0.29	0.64	0.39	0.47	0.72

Local richness, or α diversity, potentially provides important insights into patterns of early tetrapod diversification, as α diversity estimates may be less strongly affected by biases in sampling that can confound global diversity compilations [46]. We found that local richness for both non-amniotes and amniotes increased across the interval (figure 1*a*–*c*), contrary to the pattern recovered in previous analyses [15]. Local richness rose slowly through the Carboniferous, with most collections (=fossil localities) containing fewer than 10 species. At the end of the early Permian, this increase accelerates as the number of species per collection increases. Exceptionally well-sampled sites can be clearly seen to be isolated from the general pattern (figure 1*a–c*), further exemplifying uneven sampling during this interval. For example, exceptional sites occur in the Moscovian (Linton Diamond coal mine, Ohio and Nyrañy coal mine, Czech Republic), and Artinskian/Kungurian (Coffee Creek locality, Texas and Richard's Spur quarry site, Oklahoma) (see electronic supplementary material, table S1).

**Figure 3.**
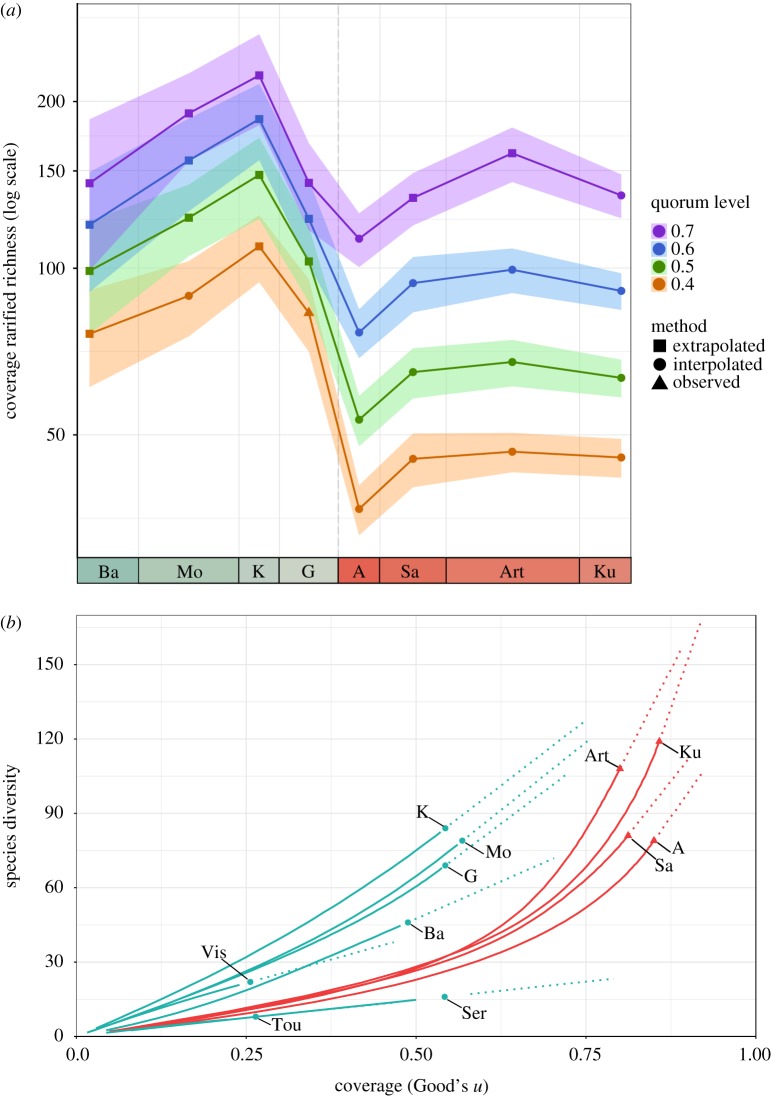
Estimates of diversity of Carboniferous–early Permian tetrapods using coverage-based subsampling. (*a*) Coverage-standardized diversity curve for intervals across the Carboniferous/Permian boundary showing estimates at different quorum levels. Abbreviations of interval names are as given in figure 1. (*b*) Coverage-based rarefaction curve for all intervals of the Carboniferous and early Permian. Extrapolated portions of lines represent analytical solutions for the Chao1 extrapolator at specific levels of coverage. Diversity was extrapolated at up to twice the reference sample size as recommended by Hsieh *et al.* [38]. Abbreviations of interval names are as in figure 1. (Online version in colour.)

Coverage-standardized richness estimates of diversity across the Carboniferous/Permian boundary suggest that diversity increased into the late Carboniferous, but fell substantially across the boundary (with the decline beginning in the Gzhelian) and subsequently began to increase again, albeit slowly, through the early Permian (figure 3*a*). However, it is important to recognize that both relative and rank-order richness can change depending on quorum level, and at higher quorum levels, the relative drop in diversity from the Carboniferous to the Permian becomes less pronounced (figure 3*b*). These estimates stand in stark contrast to the patterns of raw diversity. The marked decrease in standardized diversity across the Carboniferous/Permian boundary correlates closely with the time of the ‘rainforest collapse’, suggesting a close link between gamma diversity and floral composition. The apparent conflict between heightened local richness (alpha diversity) but lower gamma diversity in the earliest Permian relative to the late Carboniferous is explicable if beta diversity decreased (i.e. faunas became less biogeographically distinct and more cosmopolitan—as discussed below).

### (b) Patterns of biogeography

**Figure 4.**
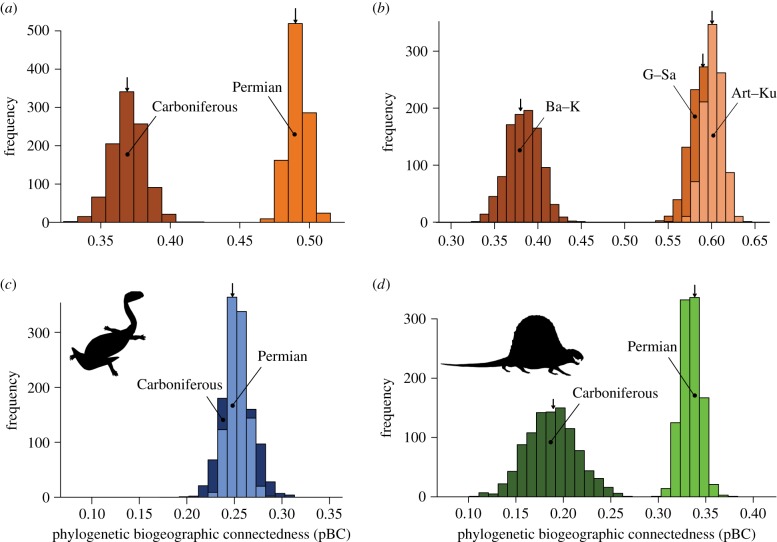
Histograms showing the distribution of bootstrap analyses of phylogenetic biogeographic connectedness (pBC). (*a*) Carboniferous and early Permian for all tetrapod species; (*b*) Bashkirian–Kasimovian (pre-CRC), Gzhelian–Sakmarian (post-CRC) and Artinskian–Kungurian (post-CRC) intervals, again for all tetrapod species; (*c*) Carboniferous and early Permian for non-amniote species; (*d*) Carboniferous and early Permian for amniote species. Higher pBC values indicate increasing connectivity between regions, and arrows indicate the mean pBC value for each interval (e.g. in (*a*) Carboniferous = 0.37, early Permian = 0.49). Abbreviations of interval names are as in figure 1. Silhouettes from phylopic.org. (Online version in colour.)

Previous investigations of early tetrapod biogeography patterns suggest that habitat fragmentation following the CRC (Kasimovian, approx. 305 Ma) drove the development of increased endemism for the first time among tetrapod faunas in the early Permian [15]. Our analyses do not support this hypothesis; instead, we recover a significant increase in global connectedness (pBC) from before the CRC (Carboniferous) to after (early Permian) (figure 4*a*). Instead of endemism developing, communities appear to have become better connected following the ‘rainforest collapse’. This same pattern is seen when three shorter intervals, instead of only two, are analysed (figure 4*b*).

Sahney *et al.* [15] formed their hypothesis of endemism based on a simple calculation of dividing global tetrapod family diversity by mean α diversity for each time bin. However, given the strong sampling biases present in the data, we argue that more sophisticated methods are necessary to decipher the responses of tetrapod faunas to the rainforest collapse. To explain their finding of enhanced endemism, Sahney *et al.* [15] invoked the theory of island biogeography [47] which suggests that habitat fragmentation can drastically affect diversity. However, this conclusion may stem from an oversimplification of the floral changes that happened at the end of the Carboniferous. Instead of the rainforests ‘collapsing’, the floral composition of the landscape at the equator transitioned gradually from wetlands to drylands [9]. The main areas of rainforests in Euramerica disappeared at the end of the Moscovian; however, areas of swamps persisted in Variscan intramontane basins in Europe and some lowland areas of central North America through the late Pennsylvanian [48,49]. Furthermore, in China, these wetland swamps did not fully develop until the late Pennsylvanian and continued to expand in the early Permian, indicating that the coal forest biome was migrating gradually eastwards during much of the late Carboniferous [49]. This change in floral composition at the end of the Carboniferous, while recorded as a mass extinction event in the plant fossil record [30], may not have resulted in tetrapod communities being isolated from one another by new, unsuitable landscape as suggested by Sahney *et al.* [15].

Instead, more open landscapes could conceivably have favoured dispersal, leading to increased connectivity between previously separate faunal communities. Amphibians do not show any significant change in biogeographic connectedness from the Carboniferous–early Permian (figure 4*c*), suggesting that dispersal rates did not increase following the disappearance of the rainforests, and that the pattern of increased connectedness in tetrapod faunas in the early Permian is driven primarily by amniotes (figure 4*d*). Amniotes, such as edaphosaurids and sphenacodontids, with their generally larger body size relative to earlier tetrapods, began to appear at the end of the Carboniferous and in early Permian. Unlike amphibians, which dominated earlier faunas, these taxa were not confined to wetland environments and could freely disperse across the new landscape.

## 4. Conclusion

Despite recent concerted attempts to close the gaps in our knowledge of early tetrapod diversity [3,50], tetrapod data for the Carboniferous and early Permian are still lacking. Nevertheless, using a newly complied species-level dataset and a range of quantitative approaches for estimating patterns of diversity and biogeography, we have been able to comprehensively test the major patterns of diversity change during this interval. Species diversity increased towards the end of the Carboniferous, before decreasing across the Carboniferous/Permian boundary and subsequently remaining lower in the early Permian. Our analyses of early tetrapod biogeography do not support the previous hypothesis that habitat fragmentation following the end-CRC drove the development of endemism, resulting in tetrapod communities diversifying in isolation in the early Permian. Instead, we found that tetrapod communities were increasingly well connected following the ‘rainforest collapse’, which may have led to lower gamma diversity. This ‘collapse’ of the rainforests is better represented as a gradual transition between wetlands and drylands, and resulted in a more open landscape which favoured dispersal, particularly among amniote faunas.

## Supplementary Material

Supplementary Information

## Supplementary Material

A Early tetrapod occurrence dataset

## Supplementary Material

B Early tetrapod species occurrences (cleaned)

## Supplementary Material

C Early tetrapod supertree

## Supplementary Material

Standardised diversity using iNEXT

## Data accessibility

Fossil occurrence data, R scripts and the tree file are available as online electronic supplementary material.
