# Supplementary material for: Diversity change during the rise of tetrapods and the impact of the ‘Carboniferous rainforest collapse’
Source: Proc Biol Sci. 2018 Feb 7;285(1872):20172730. doi: 10.1098/rspb.2017.2730 (PMC5829207; doi:10.1098/rspb.2017.2730)
Supplement: Supplementary Information [file rspb20172730supp1.docx]

**Supplementary Information**

**Diversity change during the rise of tetrapods and the impact of the ‘Carboniferous Rainforest Collapse’**

Dunne *et al.* (2018) *Proceedings of the Royal Society: B*

Corresponding author email: dunne.emma.m@gmail.com

**Outline of contents**

1. Data collection
2. Raw diversity
3. Sampling through time
4. Subsampling & iNEXT analyses
5. Sampling in the Carboniferous–early Permian
6. Phylogenetic Biogeographic Connectedness analysis
   1. Taxon occurrences and ages
   2. Geographical regions
   3. Phylogeny
7. References for supplementary information
8. **Data Collection**

Occurrences for species of Tetrapodomorpha from the Tournaisian to Kungurian (358.9–272.3 Ma) were downloaded from the Paleobiology Database (accessed via www.paleobiodb.org on September 19^th^ 2017). All data are provided as supplementary data files. Data cleaning and analyses were conducted in R (version 3.4.1). The full dataset containing all occurrences (including those which are indeterminate at species level) includes 1,582 global occurrences (supplementary data A). When the dataset was cleaned to only include valid species (n=476), the total number of occurrences was 1,047 (supplementary data B).

1. **Raw Diversity**

Global (=gamma scale) raw diversity curves were plotted using sampled-in-bin counts of specifically determinate occurrences using the function within the SQS Perl script provided by John Alroy. In order to count all taxa with ranges spanning one bin or more, the “deorphan” option was set to ‘yes’, meaning that collections spanning multiple bins were assigned to bins including more than half of their age estimate limits.

Alpha diversity (or local richness) was obtained by counting total species per collection. These counts included not only occurrences determinate at species level but also occurrences indeterminate at species level that must logically represent distinct species according to the taxonomic hierarchy of the Paleobiology Database. This allowed a more accurate picture of local diversity, using all of the available data. We present raw estimates of local richness because sampling-standardised estimates would require abundance data, which is not consistently available in the literature.

Although recent work by our group has argued that ‘global’ diversity curves are problematic due to substantial changes in the palaeogeographic spread of localities through time (e.g. Close *et al*. 2017), we argue that this is less of a concern for the Carboniferous–early Permian because the great majority of sampling of the tetrapod record is from a relatively small palaeogeographic area (palaeoequatorial regions of Laurasia). Although increases in palaeogeographic sampling do occur through time (figure 2), they are small.

1. **Sampling Through Time**

Numbers of fossiliferous formations and collections (=fossil localities) were obtained from the Paleobiology Database download. Counts for total occupied equal-area grid cells were calculated using the package ‘dggridR’ (Barnes *et al.*, 2017) in R. These grids are hexagonal and pentagonal in shape and have 50km spacings on a global map.

1. **Subsampling & iNEXT**

We standardised our diversity samples using Shareholder Quorum Subsampling (SQS; Alroy 2009, 2010a-c), a method also known in the ecological literature as ‘coverage-based rarefaction’ (Chao & Jost 2012). SQS standardises diversity samples to equal coverage of the underlying frequency distribution. Coverage is a measure of sample completeness that can be estimated using Good's *u* (Good 1953), and corresponds to the fraction of individuals in the underlying sampling pool that are made up of the species present in the sample. Standardising your diversity data to a coverage (or “quorum” level) of 0.5 will therefore tell you how many species you would expect to find, on average, in a sample of 50% of the individuals drawn at random from the underlying sampling pool or assemblage.

We implemented SQS using the R package iNEXT (Hsieh *et al*. 2016). iNEXT implements SQS using the multinomial probability formulae derived by Chao and Jost (2012), rather than the exact algorithm described by Alroy (2014). These two approaches yield identical results when subsampling or 'interpolating' (drawing down) the data. However, iNEXT also allows extrapolating the data to equal coverage using the well-known Chao1/2 formulae (Chao 1984; see Chao and Jost 2012 for further details). This allows interpolated, observed and extrapolated estimates to be seamlessly united into a single coverage-based rarefaction curve (Figure 3A). Following the recommendation of Hsieh *et al.* (2016), we only used extrapolated estimates based on extrapolated sample sizes that were less than twice as large as the true sample size.

Coverage-based rarefaction curves, in which standardised diversity is plotted as a function of coverage, show how among-assemblage differences in the shape of the abundance distribution affect relative richness at different levels of coverage. For example, a very uneven assemblage might be less diverse than a more even assemblage at low levels of coverage, but more diverse at higher levels of coverage (because most of the individuals in the uneven assemblage are made up of the most common species). Therefore, coverage-based rarefaction curves, like size-based rarefaction curves, can cross a number of times depending on how abundance distributions differ in shape. However, coverage-based rarefaction curves require smaller sample sizes to identify when crossing points occur (Chao & Jost 2012). Combining interpolated, observed and extrapolated estimates allows us to generate diversity curves at higher levels of coverage, which makes maximal use of the available data and avoids discarding information from better-sampled assemblages (Chao & Jost 2012).

1. **Sampling in the Carboniferous–early Permian**

Sampling throughout this interval is uneven both temporally and spatially. In addition to the patterns discussed in the main text we also assessed correlation between species richness and proxies for sampling. There is a weak correlation between species richness and the number of fossiliferous formations in each interval from the Tournaisian–Kungurian (R^2^ = 0.1251, p=0.166; Pearson’s r = 0.5183) (supplementary figure 1C). However, this result may be due to a small number of intervals (namely, the Moscovian, Artinskian, and Kungurian) where species richness is high relative to sampling. Exceptionally well-sampled sites contribute greatly to the total diversity in each of these intervals (supplementary table 1). Over half of the total number of species in the Moscovian come from two extensively excavated coal mines, at Linton in Ohio (Cope, 1873–75) and Nyrañy in the Czech Republic (Fritsch, 1875, 1876). Similarly, a large proportion of the total species richness in the Artinskian and Kungurian is from quarries or sites in North America that have been repeatedly studied for many years (e.g. Coffee Creek, Texas [Cope, 1882], Craddock Bonebed, Texas [Romer, 1928], Richards Spur/Dolese Brothers Quarry, Oklahoma [Evans *et al.,* 2009]). When these particularly well-sampled intervals (Moscovian, Artinskian, and Kungurian) are removed from the analysis, there is a stronger positive correlation between species richness and formation counts (R^2^ = 0.7416, p<0.01, Pearson’s r = 0.8613) (supplementary figure 1D). A moderate correlation is seen between species richness and collection (=fossil locality) count both when all intervals are analysed, and when all three exceptionally well-sampled intervals are removed (supplementary figures A-B). These results suggest the presence of a strong ‘background’ sampling signal punctuated by a few time intervals where individual sites or formations have been exceptionally well-sampled, particularly during the Carboniferous.

**Supplementary figure 1:** Correlation between species richness and total collection count (A-B) and total formation count (C-D) per interval, when all intervals are included (A and C) and when the three most well-sampled intervals (Moscovian, Artinskian, and Kungurian) are removed (B and D). Abbreviations of interval names: Tou-Tournaisian; Vis-Visean; -Ser-Serpukhovian; Bas-Bashkirian; Mos-Moscovian; Kas-Kasimovian; Gzh-Gzhelian; Ass-Asselian; Sak-Sakmarian; Art-Artinskian; Kun-Kungurian.

**Supplementary table 1:** The most speciose collections (=fossil localities) in the Paleobiology Database from the Tournaisian to Kungurian, and the method of sampling associated with each site. The total number of species per collection is shown alongside the proportion of the interval’s total diversity each collection represents.

| **Collection & location** | **Interval** | **Formation** | **Total spp.** | **% of total interval richness** | **Sampling method** |
| --- | --- | --- | --- | --- | --- |
| Coffee Creek, TX | Kungurian | Arroyo | 36 | 34.9% | Multiple expeditions |
| Craddock Bonebed, TX | Kungurian | Arroyo | 18 | 17.4% | Quarrying |
| Richard’s Spur, OK | Kungurian | Garber | 23 | 22.3% | Quarrying |
| Archer City Bonebed 1, TX | Artinskian | Putnam/Archer | 16 | 17.8% | Multiple expeditions |
| El Cobre Canyon, NM | Gzhelian | Cutler | 15 | 25.4% | Multiple expeditions |
| Nyrañy, Czech Republic | Moscovian | Kladno | 24 | 26.9% | Coal mine |
| Linton, OH | Moscovian | Upper Freeport | 33 | 37.1% | Coal mine |

**Supplementary table 2**: Pearson’s product moment correlation co-efficient for each of the counts used in raw diversity analyses (number of species, number of fossiliferous formations, number of collections (=fossil localities), and number of occupied grid cells)

|  | Total species | Total Formations | Total Collections | Total occupied 50km^2^ grid cells |
| --- | --- | --- | --- | --- |
| Total species | - | 0.5183 | 0.7643 | 0.630 |
| Total Formations | p = 0.2858 | - | 0.7346 | 0.6961 |
| Total Collections | p = 0.0038** | p = 0.01003* | - | 0.8491 |
| Total occupied 50km^2^ grid cells | p = 0.1193 | p = <0.001*** | p = 0.01734* | - |

*p = <0.05, **p=<0.01, ***p=0.001

1. **Phylogenetic Biogeographic Connectedness analysis**

We used the method of Button *et al.* (2017) which uses both geographic and phylogenetic information to quantify phylogenetic biogeographic connectedness (pBC) between regions containing tetrapod fauna. We followed the procedure outlined in Button *et al.* and below outline the details of this analysis. Example code for these analyses can be found in the supplementary data files of the Button *et al*. paper.

1. ***Taxon occurrences and ages***

Occurrence data was taken from the same dataset downloaded from the Paleobiology Database, as outlined above in section 1 (supplementary data A). From this dataset containing all occurrences of all tetrapod species from the Carboniferous–early Permian, datasets containing occurrences for non-amniotes (Tetrapodomorpha and amphibians) and amniotes (including Reptiliomorpha) were extracted.

For analyses on all tetrapods, non-amniotes, and amniotes, taxa were placed in two time bins: Carboniferous (Tournaisian–Gzhelian) and early Permian (Asselian–Kungurian). Additionally, another analysis was carried out using occurrences of all tetrapod species using three time bins: Bashkirian–Kasimovian, Gzhelian–Sakmarian, and Artinskian–Kungurian. These time bins correspond approximately with the interval before (Bashkirian–Kasimovian) and after (Gzhelian–Sakmarian and Artinskian–Kungurian) the ‘rainforest collapse’. This additional analysis also served to corroborate the broader time bins in the initial analyses above.

1. ***Geographical regions***

Palaeocoordinates for 1,047 tetrapod occurrences were taken from the occurrence dataset and binned according to the appropriate time bins outlined above. *K*-means clustering was performed on these datasets separately in R, varying *k* from 3-10. Ten-thousand replicates were performed for each analysis, with ten random starts. The performance of each was measured as the proportion of the total variance explained by the resolved clusters (the ratio of the between clusters sum of squares: total sum of squares). The best performing iteration for each value of *k* was retained for further comparison. Comparison between results of different values of *k* was principally performed on the basis of variance explained by each, with those scoring <90% being omitted from consideration. Further comparison was performed by considering following criteria: the consistency of the clusters through the time interval in question and their consistency with previously recognised biogeographic provinces. This resulted in the designation of seven clusters to use in each of the network biogeography analyses. These clusters can be seen in supplementary table 3 and supplementary figure 2.

As noted from our analyses of diversity and sampling through time, global sampling during this interval from the Carboniferous to early Permian is uneven, both temporally and spatially. However, with the inclusion of phylogenetic information, and the use of relatively broad geographical regions, interpretations made based upon these analyses quantifying biogeographic connectedness are less vulnerable to variations in sampling than those of previous workers (e.g. Sahney *et al.* 2010). The addition of geographical regions (originating in the countries India, Russia, and Brazil) in the early Permian that are not present in the Carboniferous, should logically increase the tendency towards a pattern of endemism. However, we see the opposite pattern in the early Permian where there is a trend towards cosmopolitanism.

We performed a sensitivity analysis by combining certain geographical regions (for example, B and C in the early Permian (see supplementary table 3). In each case, the number of regions did not change our results.

**Supplementary table 3**: The geographic clusters (**A-G**) as defined by *k-*means clustering of palaeocoordinates for all tetrapod occurrences from the Carboniferous (Tournaisian–Gzhelian) and early Permian (Asselian–Kungurian). Each cluster has been assigned a name in accordance with the approximate location on a modern map.

| **CARBONIFEROUS** | | | **EARLY PERMIAN** | | |
| --- | --- | --- | --- | --- | --- |
| **A** | “Eastern” USA | West Virginia (in part), Alabama, Iowa | **A** | “Mid” USA | New Mexico, Colorado, Utah |
| **B** | “Southern” USA | Texas, New Mexico, Colorado, Oklahoma (in part), Kansas (in part) | **B** | “Southern” USA 1 | Texas and Oklahoma: e.g. Garber, Belle Plains, and Admiral formations |
| **C** | “Mid” USA | Kansas (in part), West Virginia (in part), Illinois, Ohio, Pennsylvania, Oklahoma (in part) | **G** | “Southern” USA 2 | Texas and Oklahoma: e.g. Arroyo, Vale, and Hennessey formations |
| **D** | Nova Scotia & Scotland | e.g. Joggins, East Kirkton | **D** | Eastern USA  & Brazil | Including Ohio and West Virginia |
| **E** | Mainland Europe | France, Germany, Czech Republic | **E** | Europe | Czech Republic, Germany, Britain, France |
| **F** | British Isles | Ireland, England, Scotland (in part), and some localities in Nova Scotia (e.g. Point Edward) | **F** | India | (Kashmir) |
| **G** | Australia | [single locality] | **G** | Russia |  |


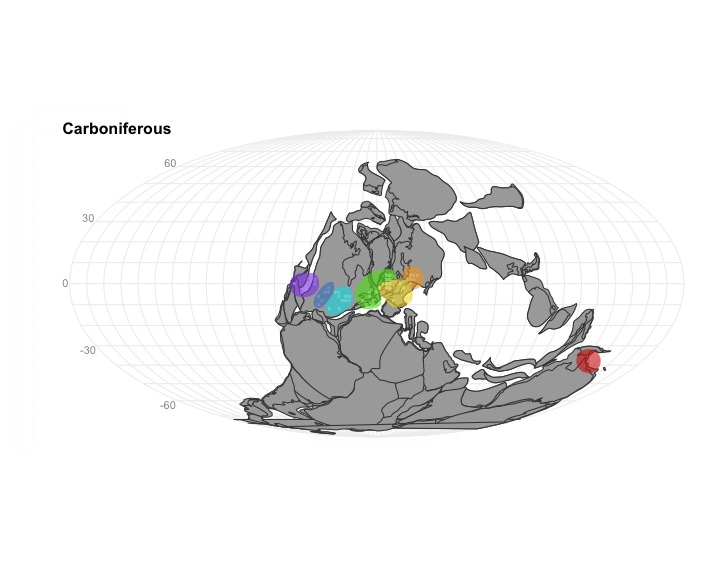


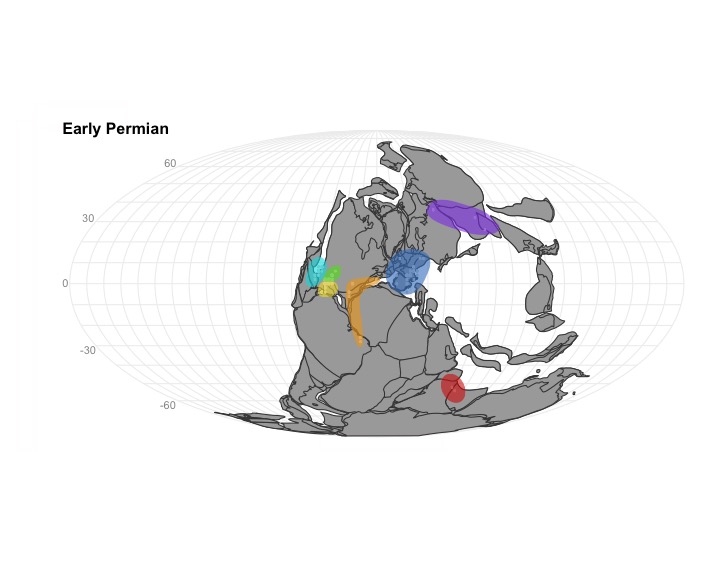


**Supplementary figure 2**: Palaeomaps illustrating the locations of the geographic regions defined by cluster analysis in both the Carboniferous and early Permian for the biogeographic analyses of all tetrapod species.

1. ***Phylogeny***

A time-calibrated informal species-level early tetrapod supertree was constructed by hand based on the most up-to-date phylogenetic analyses available for Carboniferous and early Permian terrestrial tetrapods, outlined below and in Supplementary Table 4. This tree is given as supplementary data C. This supertree includes 325 taxa within the interval from the Tournaisian to Kungurian. This topology was used to produce 100 time-calibrated trees, in which polytomies were randomly resolved, using the ‘timePaleoPhy’ function of the paleotree package in R. Trees were dated according to first occurrence dates with a minimum branch-length of 1 Myr. The phylogenetic biogeographic analyses were performed across all of these trees, in order to account for phylogenetic uncertainty.

The overall topology of the informal early tetrapod supertree follows that of Ruta *et al.* (2007) and Pardo *et al.* (2017). The phylogenetic tree of Anderson (2001) was used to place additional species of Lepospondyli on the supertree. Brocklehurst *et al.* (2015) provided a phylogenetic tree for amniotes (pelycosaurs, parareptiles, eureptiles, and therapsids) and diadectamorphs. The temnospondyl portion of the tree came from a novel “metatree” (sensu Lloyd *et al*. 2016) of temnospondyls that extends a previous formal supertree (Ruta *et al.* 2007) for Temnospondyli as a whole. Here 102 published cladistic matrices were reanalysed under a parsimony optimality criterion and all possible most parsimonious solutions encoded as matrices using Baum and Ragan (Baum 1992; Ragan 1992) Matrix Representation with Parsimony (MRP). (These are available along with the original matrices and other metadata at graemetlloyd.com/matramph.html.) Taxa were reconciled against a master list of valid species (including some as yet unnamed OTUs), with supraspecific taxa being replaced by the valid constituent species that were also present as OTUs in other source matrices. The source matrices were combined with a taxonomic hierarchy to generate a single MRP matrix, with each source matrix weighted on their dependence and publication year (see Lloyd *et al.* 2016). After searching for the optimal topologies with TNT (Goloboff *et al*. 2008) 5,248 equally parsimonious trees were returned and a strict consensus produced. This tree was subsequently pruned of younger taxa not relevant to the current analyses.

**Supplementary Table 4**: Tetrapod species included in the informal supertree

| **Early tetrapods and Tetrapodamorpha** (Ruta *et al.* 2007 and Pardo *et al.* 2017) |
| --- |
| *Colosteus scutellatus* |
| *Lethiscus stocki* |
| *Ossinodus pueri* |
| *Pederpes finneyae* |
| *Whatcheeria deltae* |
| *Crassigyrinus scoticus* |
| *Greererpeton burkemorani* |
| *Eucritta melanolimnetes* |
| *Baphetes kirkbyi* |
| *Baphetes orientalis* |
| *Baphetes planiceps* |
| **Temnospondyli** (Cashmore & Lloyd, *in prep*) |
| *Platyoposaurus watsoni* |
| *Platyoposaurus stuckenbergi* |
| *Kashmirosaurus ornatus* |
| *Amphibamus grandiceps* |
| *Doleserpeton annectens* |
| *Georgenthalia clavinasica* |
| *Gerobatrachus hottoni* |
| *Platyrhinops fritschi* |
| *Platyrhinops lyelli* |
| *Plemmyradytes shintoni* |
| *Eoscopus lockardi* |
| *Tersomius texensis* |
| *Tersomius mosesi* |
| *Pasawioops mayi* |
| *Rubeostratilia texensis* |
| *Tersomius dolesensis* |
| *Apateon caducus* |
| *Apateon dracyi* |
| *Apateon pedestris* |
| *Apateon kontheri* |
| *Apateon flagrifer* |
| *Apateon umbrosus* |
| *Apateon intermedius* |
| *Leptorophus tener* |
| *Leptorophus raischi* |
| *Schoenfelderpeton prescheri* |
| *Melanerpeton sembachense* |
| *Melanerpeton humbergense* |
| *Melanerpeton eisfeldi* |
| *Branchiosaurus gracilis* |
| *Branchiosaurus salamandroides* |
| *Branchierpeton amblystomum* |
| *Branchierpeton reinholdi* |
| *Branchierpeton saalense* |
| *Limnogyrinus elegans* |
| *Micromelerpeton ulmetense* |
| *Micromelerpeton boyi* |
| *Micromelerpeton credneri* |
| *Stegops newberryi* |
| *Cacops aspidephorus* |
| *Cacops morrisi* |
| *Conjunctio multidens* |
| *Brevidorsum profundum* |
| *Parioxys bolli* |
| *Parioxys ferricolus* |
| *Broiliellus olsoni* |
| *Dissorophus multicinctus* |
| *Broiliellus brevis* |
| *Broiliellus texensis* |
| *Broiliellus arroyoensis* |
| *Broiliellus reiszi* |
| *Aspidosaurus chiton* |
| *Aspidosaurus glascocki* |
| *Aspidosaurus novomexicanus* |
| *Aspidosaurus binasser* |
| *Platyhystrix rugosus* |
| *Acheloma cumminsi* |
| *Acheloma dunni* |
| *Phonerpeton whitei* |
| *Phonerpeton pricei* |
| *Anconastes vesperus* |
| *Tambachia trogallas* |
| *Actiobates peabodyi* |
| *Rotaryus gothae* |
| *Ecolsonia cutlerensis* |
| *Fedexia striegeli* |
| *Dendrerpeton rugosum* |
| *Dendrerpeton confusum* |
| *Dasyceps bucklandi* |
| *Dasyceps microphthalmus* |
| *Zatrachys serratus* |
| *Perryella olsoni* |
| *Dvinosaurus secundus* |
| *Dvinosaurus primus* |
| *Acroplous vorax* |
| *Isodectes obtusus* |
| *Timonya anneae* |
| *Trimerorhachis insignis* |
| *Trimerorhachis sandovalensis* |
| *Trimerorhachis greggi* |
| *Trimerorhachis rogersi* |
| *Trimerorhachis mesops* |
| *Archegosaurus decheni* |
| *Procuhy nazariensis* |
| *Neldasaurus wrightae* |
| *Eugyrinus wildi* |
| *Clamorosaurus nocturnus* |
| *Clamorosaurus borealis* |
| *Onchiodon langenhani* |
| *Onchiodon manebachensis* |
| *Onchiodon credneri* |
| *Onchiodon thuringiensis* |
| *Onchiodon labyrinthicus* |
| *Eryops grandis* |
| *Eryops megacephalus* |
| *Glaukerpeton avinoffi* |
| *Actinodon frossardi* |
| *Sclerocephalus haeuseri* |
| *Sclerocephalus jogischneideri* |
| *Sclerocephalus bavaricus* |
| *Sclerocephalus nobilis* |
| *Glanochthon angusta* |
| *Glanochthon latirostris* |
| *Archegosaurus dyscriton* |
| *Lysipterygium risinense* |
| *Intasuchus silvicola* |
| *Cheliderpeton lellbachae* |
| *Cheliderpeton vranyi* |
| *Chelydosaurus marahomensis* |
| *Syndyodosuchus tetricus* |
| *Palatinerpeton kraetschmeri* |
| *Iberospondylus schultzei* |
| *Chenoprosopus milleri* |
| *Cochleosaurus bohemicus* |
| *Cochleosaurus florensis* |
| *Adamanterpeton ohioensis* |
| *Procochleosaurus jarrowensis* |
| *Edops craigi* |
| *Capetus palustris* |
| *Balanerpeton woodi* |
| *Dendrerpeton acadianum* |
| *Caerorhachis bairdi* |
| *Silvanerpeton miripedes* |
| *Eoherpeton watsoni* |
| *Proterogyrinus scheelei* |
| *Anthracosaurus russelli* |
| *Anthracosaurus lancifer* |
| *Pholiderpeton attheyi* |
| *Pholiderpeton scutigerum* |
| *Archeria victori* |
| *Archeria crassidisca* |
| *Gephyrostegus bohemicus* |
| *Gephyrostegus watsoni* |
| *Bruktererpeton fiebigi* |
| *Utegenia shpinari* |
| *Discosauriscus austriacus* |
| *Ariekanerpeton sigalovi* |
| *Seymouria grandis* |
| *Seymouria sanjuanensis* |
| *Seymouria baylorensis* |
| *Solenodonsaurus janenschi* |
| *Westlothiana lizziae* |
| *Utaherpeton franklini* |
| *Tuditanus punctulatus* |
| *Asaphestera intermedia* |
| **Lepospondyli** (Anderson, 2001) |
| *Hapsidopareion lepton* |
| *Llistrofus pricei* |
| *Saxonerpeton geinitzi* |
| *Micraroter erythrogeios* |
| *Stegotretus agyrus* |
| *Batropetes fritschia* |
| *Batropetes appelensis* |
| *Batropetes niederkirchensis* |
| *Batropetes palatinus* |
| *Carrolla craddocki* |
| *Rhynchonkos stovalli* |
| *Cardiocephalus peabodyi* |
| *Euryodus bonneri* |
| *Euryodus dalyae* |
| *Euryodus primus* |
| *Cardiocephalus sternbergi* |
| *Sparodus validus* |
| *Odonterpeton triangulare* |
| *Hyloplesion longicostatum* |
| *Microbrachis pelikani* |
| *Acherontiscus caledoniae* |
| *Adelospondylus watsoni* |
| *Adelogyrinus simorhynchus* |
| *Oestocephalus amphiuminum* |
| *Oestocephalus granulosum* |
| *Oestocephalus vicinum* |
| *Phlegethontia linearis* |
| *Phlegethontia longissima* |
| *Ophiderpeton brownriggi* |
| *Ophiderpeton kirktonense* |
| *Ophiderpeton swisshelmense* |
| *Coloraderpeton brilli* |
| *Dolichosoma emersoni* |
| *Sillerpeton permianum* |
| *Brachydectes newberryi* |
| *Brachydectes elongatus* |
| *Lysorophus dunkardensis* |
| *Lysorophus tricarinatus* |
| *Scincosaurus crassus* |
| *Scincosaurus spinosus* |
| *Diplocaulus magnicornis* |
| *Diplocaulus salamandroides* |
| *Diplocaulus brevirostris* |
| *Diplocaulus primus* |
| *Diplocaulus recurvatus* |
| *Diploceraspis burkei* |
| *Diceratosaurus brevirostris* |
| *Keraterpeton longtoni* |
| *Keraterpeton galvani* |
| *Ptyonius marshii* |
| *Ptyonius olisthmonaias* |
| *Urocordylus wandesfordii* |
| *Lepterpeton dobbsii* |
| *Sauropleura bairdi* |
| *Sauropleura pectinata* |
| *Sauropleura scalaris* |
| **Amniotes & Diadectomorpha** (Brocklehurst et al., 2015) |
| *Limnoscelis paludis* |
| *Limnoscelis dynatis* |
| *Limnostygis relictus* |
| *Tseajaia campi* |
| *Oradectes sanmiguelensis* |
| *Orobates pabsti* |
| *Silvadectes absitus* |
| *Diadectes tenuitectes* |
| *Diadectes carinatus* |
| *Diadectes lentus* |
| *Diadectes zenos* |
| *Diadectes sideropelicus* |
| *Echinerpeton intermedium* |
| *Archaeothyris florensis* |
| *Varanosaurus acutirostris* |
| *Varanosaurus wichitaensis* |
| *Stereophallodon ciscoensis* |
| *Ophiacodon hilli* |
| *Ophiacodon major* |
| *Ophiacodon navajovicus* |
| *Ophiacodon retroversus* |
| *Ophiacodon mirus* |
| *Ophiacodon uniformis* |
| *Oedaleops campi* |
| *Vaughnictis smithae* |
| *Eothyris parkeyi* |
| *Baldwinonus trux* |
| *Baldwinonus dunkardensis* |
| *Oromycter dolesorum* |
| *Casea broilii* |
| *Casea halselli* |
| *Casea nicholsi* |
| *Trichasaurus texensis* |
| *Euromycter rutena* |
| *Angelosaurus dolani* |
| *Caseopsis agilis* |
| *Angelosaurus greeni* |
| *Cotylorhynchus romeri* |
| *Archaeovenator hamiltonensis* |
| *Apsisaurus witteri* |
| *Mycterosaurus longiceps* |
| *Ruthiromia elcobriensis* |
| *Aerosaurus greenleeorum* |
| *Aerosaurus wellesi* |
| *Varanops brevirostris* |
| *Tambacarnifex unguifalcatus* |
| *Ianthasaurus hardestiorum* |
| *Glaucosaurus megalops* |
| *Edaphosaurus credneri* |
| *Edaphosaurus microdus* |
| *Edaphosaurus mirabilis* |
| *Edaphosaurus novomexicanus* |
| *Edaphosaurus colohistion* |
| *Edaphosaurus boanerges* |
| *Edaphosaurus pogonias* |
| *Edaphosaurus cruciger* |
| *Haptodus baylei* |
| *Ianthodon schultzei* |
| *Cutleria wilmarthi* |
| *Ctenorhachis jacksoni* |
| *Secodontosaurus obtusidens* |
| *Secodontosaurus willistoni* |
| *Cryptovenator hirschbergeri* |
| *Ctenospondylus casei* |
| *Ctenospondylus ninevehensis* |
| *Bathygnathus borealis* |
| *Sphenacodon ferocior* |
| *Sphenacodon britannicus* |
| *Sphenacodon ferox* |
| *Dimetrodon angelensis* |
| *Dimetrodon booneorum* |
| *Dimetrodon dollovianus* |
| *Dimetrodon gigashomogenes* |
| *Dimetrodon grandis* |
| *Dimetrodon loomisi* |
| *Dimetrodon macrospondylus* |
| *Dimetrodon natalis* |
| *Dimetrodon occidentalis* |
| *Dimetrodon teutonis* |
| *Dimetrodon milleri* |
| *Dimetrodon limbatus* |
| *Tetraceratops insignis* |
| *Microleter mckinzieorum* |
| *Eudibamus cursoris* |
| *Bolosaurus grandis* |
| *Bolosaurus striatus* |
| *Bolosaurus major* |
| *Belebey augustodunensis* |
| *Coelostegus prothales* |
| *Thuringothyris mahlendorffae* |
| *Concordia cunninghami* |
| *Romeria prima* |
| *Romeria texana* |
| *Protocaptorhinus pricei* |
| *Reiszorhinus olsoni* |
| *Rhiodenticulatus heatoni* |
| *Captorhinus laticeps* |
| *Captorhinus aguti* |
| *Captorhinus magnus* |
| *Captorhinikos chozaensis* |
| *Captorhinikos parvus* |
| *Captorhinikos valensis* |
| *Labidosaurus hamatus* |
| *Labidosaurikos meachami* |
| *Rothianiscus multidonta* |
| *Brouffia orientalis* |
| *Paleothyris acadiana* |
| *Protorothyris morani* |
| *Protorothyris archeri* |
| *Spinoaequalis schultzei* |
| *Petrolacosaurus kansensis* |
| *Araeoscelis gracilis* |
| *Araeoscelis casei* |

**7. References**

Alroy J. 2010a Fair sampling of taxonomic richness and unbiased estimation of origination and extinction rates. In Quantitative Methods in Paleobiology, pp 55–80. Paleontological Society Short Course.

Alroy J. 2010b Geographical, environmental and intrinsic biotic controls on Phanerozoic marine diversification. *Palaeontology* **53**, 1211–1235.

Alroy J. 2010c The shifting balance of diversity among major marine animal groups. *Science* **329**, 1191–1194.

Alroy J. 2014. Accurate and precise estimates of origination and extinction rates. *Paleobiology* **40**, 374–397.

Anderson JS. 2001 The phylogenetic trunk: maximal inclusion of taxa with missing data in an analysis of the Lepospondyli (Vertebrata, Tetrapoda). *Syst. Biol*. **50**, 170–193.

Barnes R, Sahr K, Evenden G, Johnson A, Warmerdam F. 2017 dggridR: Discrete Global Grids for R. Version 2.0.1. URL: <https://github.com/r-barnes/dggridR/>

Baum BR 1992 Combining trees as a way of combining data sets for phylogenetic inference, and the desirability of combining gene trees. *Taxon* **41**, 3–10.

Brocklehurst N, Ruta M, Müller J, Fröbisch J. 2015 Elevated extinction rates as a trigger for diversification rate shifts: early amniotes as a case study. *Sci. Rep*. **5**, 17104.

Chao A, Jost L. 2012 Coverage-based rarefaction and extrapolation: Standardizing samples by completeness rather than size. *Ecology* **93**, 2533–2547.

Close RA, Benson RBJ, Upchurch P, Butler RJ. 2017 Controlling for the species-area effect supports constrained long-term Mesozoic terrestrial vertebrate diversification. *Nature Communications* **8**, 15381.

Cope ED. 1873 On some new Batrachia and fishes from the Coal Measures of Linton, Ohio. *Proceedings of the Academy of Natural Science of Philadelphia*340-343.

Cope ED. 1874 Supplement to the extinct Batrachia and Reptilia of North America. I. Catalogue of the air-breathing Vertebrata from the Coal-measures of Linton, Ohio. *Transactions of the American Philosophical Society***15**, 261-278.

Cope ED. 1875 Supplement to the Extinct Batrachia and Reptilia of North America I. Catalogue of the Air Breathing Vertebrata from the Coal Measures of Linton, Ohio. *Transactions of the American Philosophical Society*, New Series **15**, 261-278.

Cope ED. 1882. Third contribution to the history of the Vertebrata of the Permian formation of Texas. *Proceedings of the American Philosophical Society***20**: 447-461

Evans DC, Maddin HC, Reisz RR. 2009 A Re-Evaluation of Sphenacodontid Synapsid Material from the Lower Permian Fissure Fills near Richards Spur, Oklahoma. *Palaeontology***52**, 219-227.

Fritsch A. 1875 Uber die Fauna der Gaskohle des Pilsner und Rakonitzer Beckens. *Sitzungs-Berichte der koniglichen bohmischen Gellschaft der Wissenschaften* Prag 70-79.

Fritsch A. 1876 Über die Fauna der Gaskohle des Pilsner und Rakonitzer Beckens. *Sitzungs-Berichte der koniglichen bohmischen Gellschaft der Wissenschaften Prag* 70-78.

Goloboff PA, Farris JS, Nixon KC 2008 TNT, a free program for phylogenetic analysis. *Cladistics* **24**, 774-786.

Good IJ 1953 The population frequencies of species and the estimation of population. *Biometrika*, **40**, 237–264.

Hsieh TC, Ma KH, Chao A. 2016 iNEXT: an R package for rarefaction and extrapolation of species diversity (Hill numbers). *Methods Ecol Evol.* **7**, 1451–1456. (doi: 10.1111/2041-210X.12613)

Lloyd GT, Bapst DW, Friedman M Davis KE 2016 Probabilistic divergence time estimation without branch lengths: dating the origins of dinosaurs, avian flight, and crown birds. *Biol. Lett.,* **12**, 20160609.

Pardo JD, Szostakiwskyj M, Ahlberg PE, Anderson JS. 2017 Hidden morphological diversity among early tetrapods. *Nature* **546**, 642-645. (doi:10.1038/nature22966)

Ragan M. 1992 Phylogenetic inference based on matrix representation of trees. *Molecular Phylogenetics and Evolution*, **1**, 113-126

Romer AS 1928 Vertebrate faunal horizons in the Texas Permo-Carboniferous red beds. *University of Texas Bulletin*2801, 67-108.

Ruta M, Coates MI 2007 Dates, nodes and character conflict: Addressing the Lissamphibian origin problem. J. Sys. *Palaeontology* **5**, 69-122.

Ruta M, Pisani D, Lloyd GT, Benton MJ 2007 A supertree of Temnospondyli: cladogenetic patterns in the most species-rich group of early tetrapods. *Proc. R. Soc. B,* **274**, 3087-3095.
